# Supplementary material for: Genome-wide CRISPR Screens in T Helper Cells Reveal Pervasive Crosstalk between Activation and Differentiation
Source: Cell. 2019 Feb 7;176(4):882–896.e18. doi: 10.1016/j.cell.2018.11.044 (PMC6370901; doi:10.1016/j.cell.2018.11.044)
Supplement: Data S2. Processed Data from All the Steps of the Analysis, Related to Figure 1 [file mmc2.zip › supplemental data/motif analysis/Stat6_SRX021632_homer/homerResults/motif14.similar.html]

motif14

## Information for motif14

C
T
A
G
A
G
T
C
A
G
T
C
C
G
T
A
C
G
T
A
A
C
G
T
T
A
G
C
T
C
G
A
A
T
C
G
T
G
C
A
  
Reverse Opposite:  

A
C
G
T
T
A
G
C
A
G
C
T
A
T
C
G
C
G
T
A
A
C
G
T
A
C
G
T
A
C
T
G
A
C
T
G
A
G
T
C
  

|  |  |
| --- | --- |
| p-value: | 1e-124 |
| log p-value: | -2.872e+02 |
| Information Content per bp: | 1.724 |
| Number of Target Sequences with motif | 1714.0 |
| Percentage of Target Sequences with motif | 6.78% |
| Number of Background Sequences with motif | 881.1 |
| Percentage of Background Sequences with motif | 3.65% |
| Average Position of motif in Targets | 98.0 +/- 55.1bp |
| Average Position of motif in Background | 93.0 +/- 63.5bp |
| Strand Bias (log2 ratio + to - strand density) | 0.1 |
| Multiplicity (# of sites on avg that occur together) | 1.16 |
| Motif File: | file (matrix) reverse opposite |

### Similar de novo motifs found

|  |  |  |  |  |  |  |  |
| --- | --- | --- | --- | --- | --- | --- | --- |
| Rank | Match Score | Redundant Motif | P-value | log P-value | % of Targets | % of Background | Motif file |
| 1 | 0.922 | C T G A C T A G A G T C A G T C C G T A C G T A A C G T T A G C T C G A T A C G T G C A C T A G | 1e-109 | -252.451252 | 6.41% | 3.54% | motif file (matrix) |
| 2 | 0.916 | C T G A C T A G A G T C A T G C C G T A C G T A A C G T T A G C | 1e-106 | -244.307176 | 7.23% | 4.20% | motif file (matrix) |
